# Supplementary material for: White matter tracts adjacent to the human cingulate sulcus visual area (CSv)
Source: PLoS One. 2024 Apr 5;19(4):e0300575. doi: 10.1371/journal.pone.0300575 (PMC10997140; doi:10.1371/journal.pone.0300575)
Supplement: S2 Fig — Comparison between A. all of CSv streamlines generated using ensemble tractography [44] and B. CSv streamlines that remained after the application of LiFE [41, 57] in the left hemispheres of four subjects (S1-S4). Left CSv (yellow) identified by fMRI is shown together with streamlines (light grey) terminating near CSv. CSv and streamlines are overlaid on the sagittal (left), coronal (middle), and axial (right) sections of T1-weighted image. (PDF) [file pone.0300575.s002.pdf]

Supporting information for

White matter tracts adjacent to the human cingulate sulcus visual area (CSv)

Maiko Uesaki[1,2,3*], Michele Furlan[4], Andrew T. Smith[5], Hiromasa Takemura[1,6,7*]

1. Center for Information and Neural Networks (CiNet), Advanced ICT Research Institute, National Institute of Information and Communications Technology (NICT), Suita, Osaka, Japan
2. Graduate School of Frontier Biosciences, Osaka University, Suita, Osaka, Japan
3. Open Innovation & Collaboration Research Organization, Ritsumeikan University, Ibaraki, Osaka, Japan
4. Scuola Internazionale Superiore di Studi Avanzati (SISSA), Trieste, Trieste, Italy
5. Department of Psychology, Royal Holloway, University of London, Egham, Surrey, UK
6. Division of Sensory and Cognitive Brain Mapping, Department of System Neuroscience, National Institute for Physiological Sciences, Okazaki, Aichi, Japan
7. Graduate Institute for Advanced Studies, SOKENDAI, Hayama, Kanagawa, Japan

Corresponding authors:
Maiko Uesaki
Center for Information and Neural Networks (CiNet), National Institute of Information and Communications Technology (NICT)
E-mail: uesaki@nict.go.jp

Hiromasa Takemura
National Institute for Physiological Sciences
E-mail: htakemur@nips.ac.jp

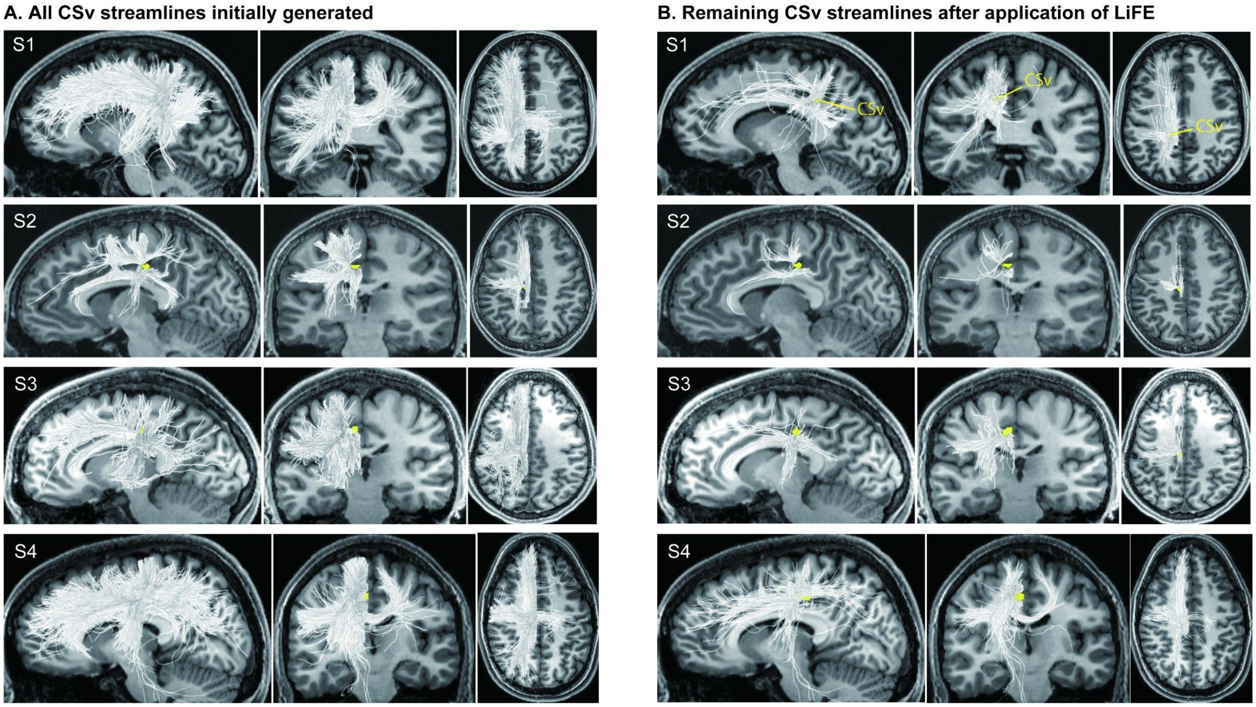

S2 Fig. Comparison between A. all of CSv streamlines generated using ensemble tractography [44] and B. CSv streamlines that remained after the application of LiFE [41,57] in the left hemispheres of four subjects (S1-S4). Left CSv (yellow) identified by fMRI is shown together with streamlines (light grey) terminating near CSv. CSv and streamlines are overlaid on the sagittal (left), coronal (middle), and axial (right) sections of T1-weighted image.
