# Supplementary material for: White matter tracts adjacent to the human cingulate sulcus visual area (CSv)
Source: PLoS One. 2024 Apr 5;19(4):e0300575. doi: 10.1371/journal.pone.0300575 (PMC10997140; doi:10.1371/journal.pone.0300575)
Supplement: S3 Fig — A. Streamlines not categorised as part of major white matter tracts in the left hemisphere of one representative subject (S1). Conventions are identical to those in Fig 4. B. Short-range streamlines connect CSv with the superior parietal regions. Left CSv (yellow) and the trajectories of streamlines that were not categorised as part of major white matter tracts (orange) are overlaid on a representative coronal section of the T1-weighted volume. Some of the streamlines can be observed connecting CSv and the superior parietal regions, presumably including the precuneus and the superior parietal lobule. (PDF) [file pone.0300575.s003.pdf]

Supporting information for

White matter tracts adjacent to the human cingulate sulcus visual area (CSv)

Maiko Uesaki[1,2,3*], Michele Furlan[4], Andrew T. Smith[5], Hiromasa Takemura[1,6,7*]

1. Center for Information and Neural Networks (CiNet), Advanced ICT Research Institute, National Institute of Information and Communications Technology (NICT), Suita, Osaka, Japan
2. Graduate School of Frontier Biosciences, Osaka University, Suita, Osaka, Japan
3. Open Innovation & Collaboration Research Organization, Ritsumeikan University, Ibaraki, Osaka, Japan
4. Scuola Internazionale Superiore di Studi Avanzati (SISSA), Trieste, Trieste, Italy
5. Department of Psychology, Royal Holloway, University of London, Egham, Surrey, UK
6. Division of Sensory and Cognitive Brain Mapping, Department of System Neuroscience, National Institute for Physiological Sciences, Okazaki, Aichi, Japan
7. Graduate Institute for Advanced Studies, SOKENDAI, Hayama, Kanagawa, Japan

Corresponding authors:
Maiko Uesaki
Center for Information and Neural Networks (CiNet), National Institute of Information and Communications Technology (NICT)
E-mail: uesaki@nict.go.jp

Hiromasa Takemura
National Institute for Physiological Sciences
E-mail: htakemur@nips.ac.jp

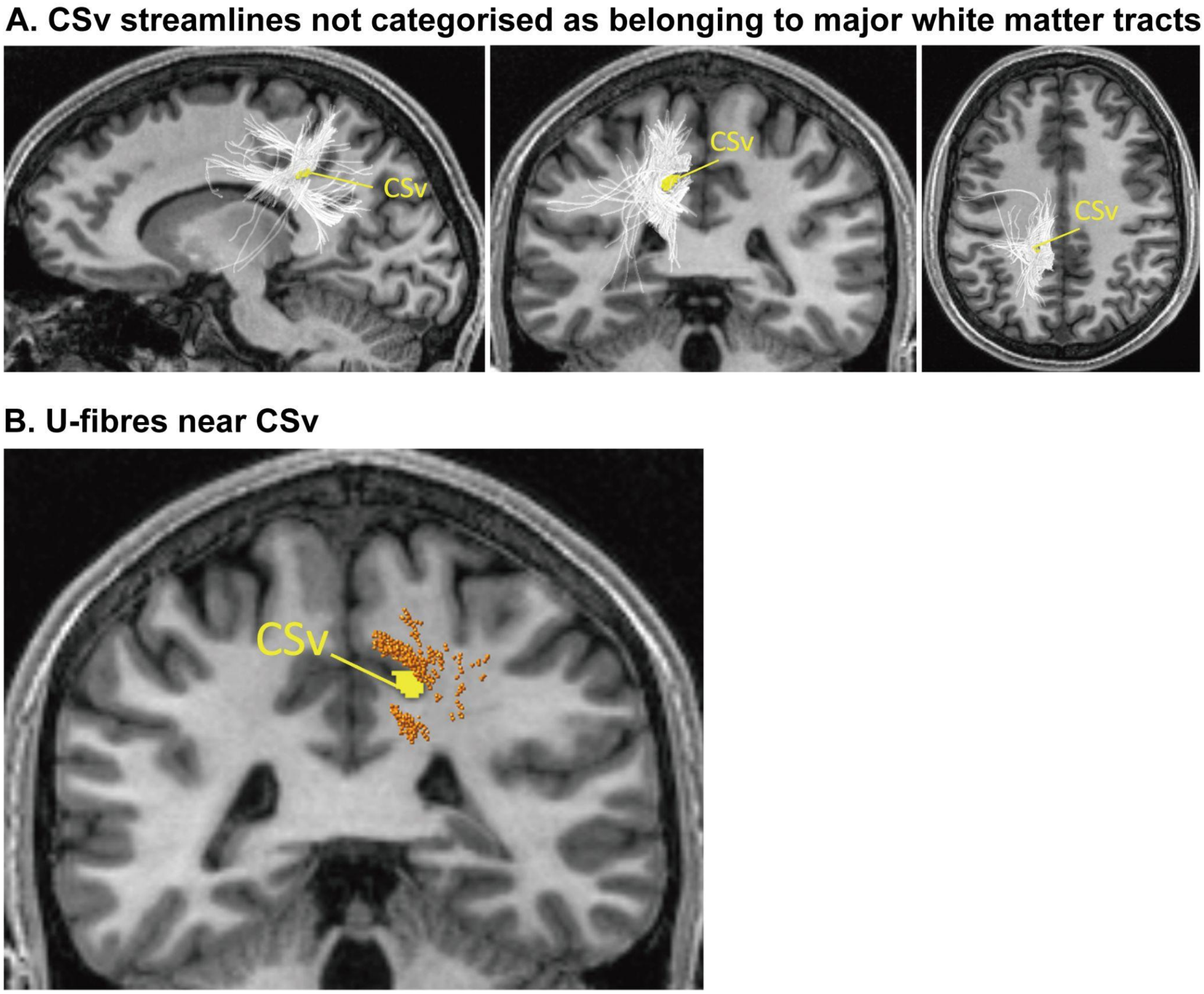

S3 Fig. CSv streamlines not categorised as part of major white matter tracts. A. Streamlines not categorised as part of major white matter tracts in the left hemisphere of one representative subject (S1). Conventions are identical to those in Fig 4. B. Short-range streamlines connect CSv with the superior parietal regions. Left CSv (yellow) and the trajectories of streamlines that were not categorised as part of major white matter tracts (orange) are overlaid on a representative coronal section of the T1-weighted volume. Some of the streamlines can be observed connecting CSv and the superior parietal regions, presumably including the precuneus and the superior parietal lobule.
