# Supplementary material for: White matter tracts adjacent to the human cingulate sulcus visual area (CSv)
Source: PLoS One. 2024 Apr 5;19(4):e0300575. doi: 10.1371/journal.pone.0300575 (PMC10997140; doi:10.1371/journal.pone.0300575)
Supplement: Attachment — Submitted filename: Response to reviewers_PLOSONE_R1.docx [file pone.0300575.s004.docx]

Dear Dr. Ogawa,

We would like to thank the editors and two anonymous reviewers for their constructive comments, which have helped us to improve our manuscript. Below, we address all comments point-by-point, and outline subsequent revisions to the manuscript. We marked the comments in the decision letter in *blue* and the changes from our original submission in red.

**Reviewer #1**

*In this manuscript, Uesaki and colleagues use diffusion tractography data to ask about the white matter used by streamlines of the human cingulate sulcus visual area (CSv). In a previous work, Smith et al. established the location of the CSv in individual subjects using  a functional localizer, then examined the endpoints of streamlines originating there (as well as various functional connectivity properties). Here, using the same dataset, the streamlines are categorized according to known white matter bundles. They mainly show CSv usage of the SLF I and cingulum.*

*This is a relatively straightforward contribution to the literature; although not a major advance, it is potentially a useful one, and certainly deserves publication. I do think there is a little work to do to really understand how these fibers travel.*

We thank the reviewer for this summary and careful assessment of our manuscript. We respond to each comment point-by-point below.

*The biggest problem is that 75% of the streamlines are unaccounted for. I understand that the authors think that these represent short-range connections, and that their validity is somewhat in question, but more work needs to be done on this problem. I have a few suggestions. First, I think we need to see these streamlines on their own. In other words, essentially reproduce Figure 5 but remove the categorized fibers (the ones belonging to SLF I, cingulum, CC, etc). Then show additional (coronal, horizontal) views to figure out what these uncategorized fibers really are. If they are entirely short-range fibers, some of them are probably quite accurate; after all, cortical regions tend to connect extremely strongly with nearby regions. There may be long-range fibers in there, too, though. It’s possible that some of them ought to be categorized as one of the known bundles, but have been left out by the particular approach used here.*

We thank the reviewer for this suggestion. In the revised manuscript, we include a new supplementary figure visualising CSv streamlines that were not categorised into major tracts (S3A Fig).

We note that amongst these streamlines, the most prominent were those connecting between CSv and the more superior regions (S3B Fig). These streamlines are likely part of the U-fiber surrounding the cingulate sulcus. The other site these streamlines mostly terminate appear more posterior, presumably including the precuneus and the superior parietal lobule. However, we prefer not to discuss these streamlines extensively in the Results section, since this is a qualitative analysis without a ground of known underlying neuroanatomy. Instead, we include a statement about these streamlines in the Discussion section in the revised manuscript.

**Specific changes:** We added a supplementary figure (S3 Fig).

**S3 Fig. CSv streamlines not categorised as part of major white matter tracts.** A. Streamlines not categorised as part of major white matter tracts in the left hemisphere of one representative subject (S1). Conventions are identical to those in Fig 4. B. Short-range streamlines connect CSv with the superior parietal regions. Left CSv (yellow) and the trajectories of streamlines that were not categorised as part of major white matter tracts (orange) are overlaid on a representative coronal section of the T1-weighted volume. Some of the streamlines can be observed connecting CSv and the superior parietal regions, presumably including the precuneus and the superior parietal lobule.

In Results, we wrote (pg. 14):

We then categorised the streamlines terminating around CSv, based on the waypoint ROIs through which they passed, to evaluate the relationship between CSv streamlines and the major white matter tracts (Fig 2; see S3 Fig for CSv streamlines not categorised into major white matter tracts).

In Discussion, “Conclusions and future directions”, we wrote (pg. 22):

In this study, we focussed on CSv streamlines that belong to major white matter tracts, rather than the rest including short-range streamlines, since it is difficult to classify streamlines that do not belong to white matter tracts known to exist in anatomical studies. For example, we observed streamlines connecting CSv and the part of the parietal cortex that likely includes the precuneus and the superior parietal lobule (S3B Fig). While these streamlines are of interest as some of the regions involved in optic-flow processing are located in this general area [9, 10, 30], we did not include these streamlines in the main analysis as short-range streamlines are particularly susceptible to partial voluming within the grey matter and to complex fibre crossing [83] and therefore we have limited confidence in the short-range CSv streamlines identified in this study using dMRI data with a standard resolution.

*Second, I am looking at Figure 5 and comparing it to the endpoints in Smith et al., and they just really don’t match up. I think that the authors of the current study are somehow throwing away a lot of connectivity identified in Smith et al. Why is that? I don’t see any or many streamlines that could account for the substantial connectivity shown in the earlier paper with V1/V2/V3 or MT, in particular. One solution here would be to look at the endpoints of all the fibers, and then also the fibers categorized as belonging to the known white matter bundles.*

The number of streamlines analysed in the present study is less than that in the previous paper (Smith et al., 2018) because we used a more conservative approach and removed streamlines that did not account for diffusion signals in each voxel. In general, it is well known that probabilistic tractography is highly sensitive, which makes it prone to producing spurious streamlines that do not align with the orientations of diffusion signals along the trajectories. To control for this, a number of filtering methods have been proposed, which cull the streamlines that do not explain diffusion signals (Pestilli et al., 2014; Dadduci et al., 2015; Smith et al. 2015). In the present study, we used a method, termed Ensemble Tractography (Takemura et al., 2016), which is an extension of one of these filtering methods (Linear Fascicle Evaluation, LiFE; Pestilli et al., 2014). We aimed to obtain more conservative results by (1) focussing on known white matter tracts and (2) discarding streamlines that do not account for diffusion signals.

In the revised manuscript, we include a new Supplementary Figure (S2 Fig) which compares CSv streamlines with and without application of LiFE. We found that streamlines travelling from CSv to the visual cortex such as V1, as suggested by the reviewer, can be observed in the original set of streamlines before LiFE was applied; but they can not be seen after application of LiFE. This does not mean that these streamlines do not exist, but indicates that these streamlines were not justified by our dMRI data.

We also revised the main text. Firstly, we explain more thoroughly why we applied the filtering to the initially generated set of streamlines in the Introduction and Materials and Methods sections. Secondly, we describe the results of comparing CSv streamlines with and without application of LiFE in the Results section. Finally, we outline considerations for the choice of tractography method in the Discussion section.

**Specific changes:** We added a new supplementary figure (S2 Fig).

**S2 Fig. Comparison between A. all of CSv streamlines generated using ensemble tractography [44] and B. CSv streamlines that remained after the application of LiFE [41,57] in the left hemispheres of four subjects (S1-S4).** Left CSv (yellow) identified by fMRI is shown together with streamlines (light grey) terminating near CSv. CSv and streamlines are overlaid on the sagittal (left), coronal (middle), and axial (right) sections of T1-weighted image.

In Introduction, we added (pg. 4):

We also applied filtering to generated streamlines, which resulted in more conservative estimates of structural connectivity supported by the dMRI data [41, 42, 43, 44].

In Materials and Methods, “4. dMRI data acquisition and analysis”, “3. Fibre tracking and evaluation”, we revised the following paragraph (pg. 8):

We used the ensemble tractography method [44]. This minimises the known dependency of tractography on the choice of parameters [53, 54, 55, 56] by generating streamlines using multiple tractography parameters. Amongst the streamlines generated, those that did not align with diffusion signals along the trajectories were then culled using linear fascicle evaluation (LiFE; [41, 57]; <http://francopestilli>.github.io/life/), which is one of the methods developed to filter and remove spurious streamlines. Our approach aimed to achieve a more conservative selection of streamlines, while reducing the dependency of tractography on arbitrary choices of parameters.

Specifically, we generated eight million candidate streamlines using CSD-based probabilistic tractography implemented in MRtrix3 (iFOD2; [58]) using four angle thresholds (5.7, 11.5, 23.1, and 47.2 deg; two million candidate streamlines per angle threshold). We set the minimum streamline length at 4 mm and the maximum at 250 mm. The default parameters of MRTrix3 CSD-based probabilistic tractography were used except for the maximum streamline length (250 mm). The seed voxels for tracking were randomly chosen from the grey-white matter interface region [59]. Finally, LiFE; [41, 57]) was applied to filter streamlines. This process removed streamlines that did not contribute to predicting diffusion signals and therefore yielded the optimal set of streamlines (optimised streamlines). In subsequent analyses, we considered only the optimised streamlines. Technical details of the ensemble tractography method are described in previous works [26, 44, 60].

In Results, we wrote (pg. 14):

We note that we took a conservative approach and analysed only the optimised streamlines (see S2 Fig for a comparison between CSv streamlines with and without application of LiFE).

In Discussion, “Conclusions and future directions”, we wrote (pg. 23):

It should also be noted that we took a relatively conservative tractography approach in the present study, applying a filter to the whole-brain streamlines initially generated in order to cull the streamlines that did not account for diffusion signals [41, 44, 57]. It is well established that probabilistic tractography is prone to producing spurious streamlines. Filtering the streamlines has been shown to yield more reliable estimates of structural connectivity [41, 44, 88, 89]. This process removed streamlines including, for example, those connecting CSv and the visual cortex, which were present amongst the streamlines that were initially generated (S2 Fig). This does not necessarily mean that structural connections do not exist between CSv and the visual cortex, however, these streamlines were not sufficiently supported by our dMRI data according to the conservative criteria of the filtering process. As discussed above, it is possible that if streamlines that contribute to genuine connections have been filtered out, similar streamlines may survive the same filtering process if generated from future dMRI data acquired with improved methods.

*Third, it seems like there are known bundles, especially the middle longitudinal fasciculus and the various dorsal-ventral bundles I’m sure this group is quite familiar with, that should be part of this analysis.*

*These three comments are related. Basically, I think that there are likely connections that need to be accounted for in one way or another either explain why they are not there, or categorize them more carefully.*

We thank the reviewer for raising this point. Accordingly, we have revised the Materials and methods section to clarify our criteria for selecting tracts of interest in this study. We referenced two established atlases of white matter tracts (Schmahmann & Pandya 2006 for macaques; Catani & Thiebaut de Schotten 2012 for humans) in our selection process. We selected the tracts based on their proximity to CSv, which is located in the posterior part of the mid-cingulate sulcus, according to the descriptions of these tracts in the atlases. We would like to note here that the middle longitudinal fasciculus (MdLF) does not satisfy our criterion since it connects the superior temporal gyrus and inferior parietal lobule (Makris et al. 2009, Cerebral Cortex), without travelling near the mid-cingulate sulcus where CSv is located. In fact, previous work has placed a NOT ROI in the region near CSv (Wang et al. 2013, Cerebral Cortex).

Based on this criterion, we have additionally included the arcuate fasciculus (AF) and corticospinal tract (CST) in the analyses.

**Specific changes:** Figure 6 (Figure 7 in the original manuscript) now includes results for AF and CST in addition to SLF I, II, III, the cingulum and the callosal fibres.

**Fig 6. Summary statistics of the proportion of CSv streamlines belonging to each of the major white matter tracts considered in the left (blue) and right (yellow) hemispheres.** CSv streamlines belonging to SLF I and the cingulum together accounted for approximately 18% of all CSv streamlines. Error bars depict ±1 SEM across subjects. SLF: superior longitudinal fasciculus, AF: arcuate fasciculus, CST: corticospinal tract.

In Materials and Methods, “5. Identification of white matter tracts adjacent to CSv”, we wrote (pg. 10):

We selected seven candidate major white matter tracts (SLF I, II, III, the cingulum, the callosal fibres, the arcuate fasciculus, and the corticospinal tract), whose proximity to CSv suggests that they could be associated with CSv, by comparing the coordinates of CSv and the positions of major white matter tracts described in the established atlases of the white matter [31, 33]. We used the waypoint ROI approach to segment subsets of CSv streamlines belonging to the major white matter tracts (Fig 2), following the criteria as in previous work [35, 37, 40, 61].

In Materials and Methods, “5. Identification of white matter tracts adjacent to CSv”, we also added two new sections (pg. 12):

### 4. Arcuate fasciculus

We identified CSv streamlines turning into the arcuate fasciculus (AF) using a waypoint ROI automatically generated by AFQ MATLAB toolbox [40]. The waypoint ROI covered an area of the white matter in the axial slice between the temporal and the fronto-parietal cortices [64]. CSv streamlines passing through this ROI were defined as belonging to AF.

### 5. Corticospinal tract

We identified CSv streamlines turning into the corticospinal tract (CST) using two waypoint ROIs automatically generated by AFQ MATLAB toolbox [40]. The inferior ROI was placed in the axial slice of the brainstem and covered the entire cerebral peduncle. The superior ROI was placed in the axial slice and covered an area of the white matter composed predominantly of tracts with a superior-inferior trajectory, located inferior to the region where CST and callosal fibres cross each other. CSv streamlines passing through both of these ROIs were defined as belonging to the CST.

*Minor comment*

*- I don’t think Figure 1 is adding much; I would not include it, but would just refer the reader back to Wall and Smith. As the first figure the reader encounters, it just isn’t that relevant.*

We agree with the reviewer. We have removed this figure in the revised manuscript. Please note that, accordingly, the numbers assigned to remaining figures have shifted.

**Specific change:** Figure 1 of the original manuscript has been omitted.

**Reviewer #2**

*The authors examined the white matter tracks based on diffusion magnetic resonance imaging (dMRI) of 12 healthy young people. The goal was to test the spatial correspondence of tracks connected with the cingulate sulcus visual (CSv) area, an region involved in visual-*

*vestibular processing, with known white matter tracks in the dorsal brain. The authors found that CSv tracks corresponded most strongly with the superior longitudinal fasciculus I (SLF I), the cingulum track, and callosal tracks, but not or less with the SLF II, SLF III.*

*The study reanalyzes data that was already published (as mentioned in text), but with a different analysis approach. Nevertheless, the findings substantially elaborate this previous work and hence are sufficiently novel and relevant. The analysis is solid. However, some aspects of the manuscript should be elaborated.*

We thank the reviewer for this summary and overall positive assessment of our manuscript. We respond to each comment point-by-point below.

*Abstract: Please do not cite articles in the abstract, because it may be ambiguous, if no full reference is given. Referring to 'published data' would be sufficient in the abstract. Citing the article in text (introduction, methods, discussion) is necessary and sufficient.*

We thank the reviewer for this suggestion. In the revised manuscript, we include the full reference to remove the ambiguity of this citation. We are happy to remove it if the editor and/or publisher consider that a citation appearing in the abstract is not appropriate, considering the journal guidelines.

**Specific changes:** In Abstract, we added (pg. 1):

A previous neuroimaging study (Smith et al. 2018, Cerebral Cortex, 28, 3685-3596) used diffusion-weighted magnetic resonance imaging (dMRI) to examine the structural connectivity of CSv, and demonstrated connections between CSv and the motor and sensorimotor areas in the anterior and posterior cingulate sulcus.

*l. 111ff: More details on known tracts is needed. This paragraph needs to be elaborated. For instance, what were the criteria for tract classification? How do the different tract categorization schemes differ or agree? How many tracts do the cited references categorize overall? What were the criteria for selecting SLF I, II, III, cingulum, and callosal fibres and ignoring others? Why were only these five tracts analyzed?*

We thank the reviewer for this comment. We have made changes to the text to clarify our criterion for selecting tracts of interest. As stated in the revised manuscript, we included tracts that are spatially close to CSv in the atlas space, based on published atlases of white matter tracts (Schmahmann & Pandya 2006 for macaques; Catani & Thiebaut de Schotten 2012 for humans). We are aware that the human brain atlas (Catani & Thiebaut de Schotten 2012) only provides approximate positions of major white matter tracts averaged across multiple brains, but it is still sufficient to determine whether a tract is located near the mid-cingulate sulcus where CSv is located. Based on this criterion, we selected seven tracts for analyses.

In the revised manuscript, we include clarification as to how we selected tracts of interest, and explain why certain tracts did not satisfy our criterion, by citing established atlases as well as other anatomical studies.

Please also refer to our response to Reviewer #1, who made a similar comment.

**Specific changes:** In Materials and Methods, “5. Identification of white matter tracts adjacent to CSv”, we wrote (pg. 10):

We then examined how CSv streamlines run into major white matter tracts. We selected seven candidate major white matter tracts (SLF I, II, III, the cingulum, the callosal fibres, the arcuate fasciculus, and the corticospinal tract), whose proximity to CSv suggests that they could be associated with CSv, by comparing the coordinates of CSv and the positions of major white matter tracts described in the established atlases of the white matter [31, 33]. We used the waypoint ROI approach to segment subsets of CSv streamlines belonging to the major white matter tracts (Fig 2), following the criteria as in previous work [35, 37, 40, 61].

*l. 233ff: The waypoint definitions for SLF, cingulum, and callosal fibres must be explained more clearly. Why did the authors define them manually? Is there an automatic procedure and why was it not used? What were the criteria for manual selection (e.g., what landmarks were used)? This needs to be described at sufficient detail that an external researcher could replicate it.*

We delineated these ROIs manually, most importantly to follow the protocol already established in previous studies (Thiebaut de Schotten et al., 2011; Metzler-Baddeley et al., 2012; Jones et al., 2013; Rojkova et al., 2016; Amemiya et al., 2021). We are aware that the automated methods are advancing. However, it is not always simple to use automated methods especially for the delineation of tracts that are spatially close to each other, like SLF I, II, and III, as their proximity makes accurate placement of the waypoint ROIs by automated methods increasingly difficult.

To address the reviewer’s concern, the figure showing the manually-drawn waypoint ROIs now include labels for sulcus landmarks used to define the waypoint ROIs (Figure 2 in the revised manuscript). We also revised the Materials and methods section in the main text, so that the protocol is described in more detail, and is more transparent to readers who might be interested in replicating the analysis.

**Specific changes:** Figure 2 (Figure 3 in the original manuscript) now marks the sulci used to identify borders of the waypoint ROIs for SLF I, II, and III**.**

**Fig 2.** **Manually delineated waypoint ROIs used to identify five of the major white matter tracts analysed in this study, superimposed on a coronal section of the T1-weighted image of one representative subject (S1).** The coloured areas depict ROIs for the five tracts (cyan, SLF I; blue, SLF II; purple, SLF III; green, cingulum; yellow, callosal fibres). AC: anterior commissure, cgs: cingulate sulcus, sfs: superior frontal sulcus, iprs: inferior precentral sulcus, lf: lateral fissure, SLF: superior longitudinal fasciculus.

In Materials and Methods, “5. Identification of white matter tracts adjacent to CSv”, we wrote (pg. 11):

Five of these tracts were manually segmented, due to difficulty in delineating neighbouring tracts using automated methods. Manual segmentation of the white matter tracts also ensured consistency with previously established protocols based on anatomical information [35, 37, 61].

In Materials and Methods, “5. Identification of white matter tracts adjacent to CSv”, we also added (pg. 11):

### 1. Superior Longitudinal Fasciculus (SLF) I, II, and III

We identified CSv streamlines turning into SLF I, II, and III using the coronal waypoint ROIs. The waypoint ROIs were manually drawn for each subject on the coronal slice with the anterior commissure (AC), following the definitions used in previous studies [35, 37, 61]. Specifically, we manually drew the waypoint ROIs, each of which covered an area in the white matter in the superior frontal, middle frontal, and precentral gyri for SLF I, II, and III, respectively, in each hemisphere (Fig 2, cyan for SLF I; blue for SLF II; purple for SLF III). We used the following sulcus landmarks to draw the waypoint ROIs: the cingulate and the superior frontal sulci defined the borders of the waypoint ROI for SLF I, the inferior precentral sulcus was used as a landmark for the border between waypoint ROIs for SLF II and III, and the lateral fissure defined the inferior border of the waypoint ROI for SLF III. We defined CSv streamlines passing through those ROIs as streamlines belonging to SLF I, II, and III, respectively.

### 2. Cingulum

Similarly, we identified CSv streamlines turning into the cingulum using a coronal waypoint ROI (Fig 2). The waypoint ROI for the cingulum was manually defined in the same coronal slice as were ROIs for SLF. We defined the coronal ROI for the cingulum in the area of the white matter adjacent to the cingulate gyrus and ventral to the cingulate sulcus, as in previous studies [62, 63]. We used the orientation of the cingulate sulcus in the coronal slice to define the border between ROIs for SLF I and the cingulum. CSv streamlines passing through this ROI were defined as part of the cingulum.

*l. 255ff: I am a bit sceptical with testing number of streamlines against zero. In fiber tracking, the number (probability) of streamlines will always be > 0 even in random data. A more careful null hypothesis should be chosen. For instance, testing the number of streamlines between two tracks implicitly takes random streamlines into account. Or the authors could test the number of streamlines for each track against the track with the least number of streamlines.*

We thank the reviewer for raising this point, and we agree that performing statistical tests comparing the number of CSv streamlines belonging to each tract of interest against zero is not an ideal approach, since it is susceptible to the existence of random streamlines and would also depend on the total number of generated streamlines.

In the revised manuscript, we report the results of statistical analysis based on the proportion, rather than the number, of CSv streamlines belonging to the tracts of interest to  all CSv streamlines in each hemisphere in each subject. This way, the figure is normalised and can theoretically be zero, unlike the number of streamlines as pointed out by the reviewer. We took this approach, instead of comparing the numbers of streamlines belonging to two tracts per the reviewer’s suggestion, as the latter would require an arbitrary selection of a control tract. Since we do not have the ground truth on which tract must be null, we think that the comparison with a control tract may not be the best suited approach for the purpose and scope of our study.

**Specific changes:** In Materials and Methods, ”6. Statistical analysis”, we wrote (pg. 13):

We evaluated the statistical significance of CSv streamlines belonging to each of the seven white matter tracts considered. We first calculated the proportion of CSv streamlines belonging to each tract. We then performed a one-sample t-test for each tract in each hemisphere. A null hypothesis of this statistical test is that the proportion of CSv streamlines belonging to each white matter tract is not different from zero. We defined a statistical significance (α) as *P* = 0.007, which is equivalent to *P* = 0.05 after Bonferroni correction for seven tracts tested for each hemisphere.

In Results, we revised (pg. 16):

To test the statistical significance of the presence of CSv streamlines belonging to each tract, we performed one-sample t-tests on the proportion of CSv streamlines belonging to each tract. The proportion of CSv streamlines categorised as SLF I was significantly above zero in the right hemisphere (t_11_ = 4.30, 95% confidence interval [CI] = 0.043 – 0.133, *P* = 0.001). We also observed a similar trend for SLF I in the left hemisphere, but the effect did not reach statistical significance when multiple comparisons across tracts were taken into account (t_11_ = 3.15, CI = 0.038 – 0.211, *P* = 0.009). The proportion of CSv streamlines belonging to the cingulum was significantly above zero in the left hemisphere (t_11_ = 3.40, CI = 0.025 – 0.116, *P* = 0.006), but did not reach statistical significance in the right hemisphere (t_11_ = 1.46, CI = -0.039 – 0.194, *P* = 0.172). The proportions of CSv streamlines belonging to the other tracts (SLF II, III, the callosal fibres, AF, and CST) were not significantly larger than zero in either hemisphere (t_11_ = 1.89, CI = -0.004 – 0.05, *P* = 0.086 for left SLF II; t_11_ = 1.16, CI = -0.010 – 0.033, *P* = 0.271 for right SLF II; t_11_ = 1.37, CI = -0.004 – 0.019, *P* = 0.199 for left SLF III; t_11_ = 2.46, CI = 0.0004 – 0.008, *P* = 0.025 for right SLF III; t_11_ = 2.90, CI = 0.005 – 0.034, *P* = 0.015 for left callosal fibres; t_11_ = 2.59, CI = 0.008 – 0.098, *P* = 0.025 for right callosal fibres; t_11_ = 1.00, CI = -5.499 – 0.002, *P* = 0.339 for left AF; t_11_ = 1.00, CI = -0.001 – 0.002, *P* = 0.339 for right CST). We note that we were unable to perform statistical tests on right AF and left CST, since none of the CSv streamlines were found to belong to these tracts in any of the subjects.

*l. 268: More detailed information on the location and size of CSv should be provided. For instance, Talairach or MNI coordinates of CSv.*

We thank the reviewer for this suggestion. In the revised manuscript, we provide the MNI coordinates of the centre of cluster that include voxels belonging to CSv of all 12 subjects for each hemisphere, as reported in the original article (Smith et al. 2018).

**Specific changes:** In Materials and methods, ”3. fMRI data acquisition and analysis”, “2. Localisation of CSv”, we added (pg. 6):

As reported in Smith et al. [30], the clusters of voxels belonging to CSv combined across all subjects had centres of gravity of [9 -24 44] and [-10 -26 41] in MNI152 space in the left and right hemispheres, respectively.

*l. 371 ff: Is it possible that the cingulum track is related to the recently described vestibular pericallosal sulcus (vPCS) region (dx.doi.org/10.1152/jn.00431.2022)? The authors should discuss whether such a relationship is possible (or alternatively why it is not possible).*

We thank the reviewer for this suggestion. We cite this important work in the Discussion section of the revised manuscript.

**Specific changes:** In Discussion, ”Conclusions and future directions”, we wrote (pg. 24):

Better understanding of the tissue properties of SLF I and the cingulum, and their relations with sensory and motor performance or disorders may provide a fuller picture of the role of CSv. Since it has been established that CSv receives not only visual but vestibular input [13, 23, 24], it may also be informative to examine the relations between these white matter tracts and vestibular areas in the medial cortex such as the vestibular pericallosal sulcus [91]. Finally, it would be beneficial for future research to investigate how lesions to SLF I and the cingulum, or individual variation of those tracts correlate with behavioural measurements on self-motion perception, locomotion, and navigation.

In Reference, we added (pg. 35):

Beer AL, Becker M, Frank SM, Greenlee MW. Vestibular and visual brain areas in the medial cortex of the human brain. Journal of Neurophysiology. 2023; 129(4): 948-962.

*Fig. 1:This figure reproduces already published work. It is not essential and paritally redundant. It could be combined with Fig. 2 as a sub-panel. I even recommend to drop this figure completely.*

We agree with the reviewer. We have omitted Figure 1 of the original manuscript entirely in the revised manuscript. Please note that, accordingly, the numbers assigned to remaining figures have shifted.

**Specific change:** Figure 1 of the original manuscript has been omitted.

*Fig. 8: Although I am a bit sceptical regarding reproducing other people's work, this figure may be acceptable. However, permission must be obtained prior to acceptance. Moreover, all abbreviations must be explained in the figure legend. The relationship to the current*

*findings must be shown more clearly. For instance, it is hard to decipher whether the dotted lines of red dots correspond to SLF I or the cingulum tract.*

*Please, be more specific. Using different colors for the two tracts might help.*

Considering the reviewer’s feedback, we have decided not to include Figure 8 of the original manuscript in the revised manuscript. Instead, the revised manuscript includes a reference to the figure and page numbers of the original figure in Schmahmann and Pandya (2006) so the readers may find it.

**Specific change:** Figure 8 of the original manuscript has been omitted.

In Discussion, “Relation to macaque studies”, we wrote (pg. 21):

In their extensive report, they document fibre pathways near the cingulate gyrus and cingulate sulcus ([31], Fig 12-3, pp. 355).

In Discussion, “Relation to macaque studies”, we deleted (pg. 21):

In addition, there are axons from SLF I travelling parallel to the cingulate sulcus and terminating at the cingulate gyrus ~~(Fig 8, red dotted lines)~~.
